# Cerebrospinal Fluid Biomarkers in Spinocerebellar Ataxia: A Pilot Study

**DOI:** 10.1155/2015/413098

**Published:** 2015-07-22

**Authors:** Ashley M. Brouillette, Gülin Öz, Christopher M. Gomez

**Affiliations:** ^1^Department of Pediatrics, Monroe Carell Jr. Children's Hospital at Vanderbilt, 2200 Children's Way, Suite 2404, Nashville, TN 37232, USA; ^2^Center for Magnetic Resonance Research, University of Minnesota, 2021 6th Street SE, Minneapolis, MN 55455, USA; ^3^Department of Neurology, AMB S237, MC 2030, The University of Chicago, 5841 S. Maryland, Chicago, IL 60637, USA

## Abstract

Neurodegenerative diseases, including the spinocerebellar ataxias (SCA), would benefit from the identification of reliable biomarkers that could serve as disease subtype-specific and stage-specific indicators for the development and monitoring of treatments. We analyzed the cerebrospinal fluid (CSF) level of tau, *α*-synuclein, DJ-1, and glial fibrillary acidic protein (GFAP), proteins previously associated with neurodegenerative processes, in patients with the autosomal dominant SCA1, SCA2, and SCA6, and the sporadic disease multiple system atrophy, cerebellar type (MSA-C), compared with age-matched controls. We estimated disease severity using the Scale for the Assessment and Rating of Ataxia (SARA). Most proteins measured trended higher in disease versus control group yet did not reach statistical significance. We found the levels of tau in both SCA2 and MSA-C patients were significantly higher than control. We found that *α*-synuclein levels were lower with higher SARA scores in SCA1 and tau levels were higher with greater SARA in MSA-C, although this final correlation did not reach statistical significance after post hoc correction. Additional studies with larger sample sizes are needed to improve the power of these studies and validate the use of CSF biomarkers in SCA and MSA-C.

## 1. Introduction

Neurodegenerative diseases are chronic disorders that are characterized by progressive death of specific nerve cells in otherwise healthy individuals, leading to a gradual loss of normal brain function. Disorders such as Alzheimer's disease (AD), Parkinson's disease (PD), and the spinocerebellar ataxias are particularly difficult to monitor in patients due to the inaccessibility of pathogenic tissues and the complexity of brain organization [1, 2]. As such, biomarkers in the cerebrospinal fluid (CSF) are uniquely poised to fill information gaps, potentially helping to elucidate pathogenic mechanisms, identify prospective drug targets, and eventually monitor therapeutic progress. Biomarkers are biological measurements that provide reproducible quantitative or semiquantitative information unique to a specific disease or group of diseases. Such markers can be measured in patient samples such as tissue, blood, or CSF and are often seen as relative increases or decreases from normal concentrations or, at times, as the appearance of a protein generally not seen in that type of sample.

Spinocerebellar ataxia (SCA) 1, SCA2, SCA6, and multiple system atrophy of the cerebellar type (MSA-C) are some of the many neurodegenerative disorders that could benefit from the discovery of relevant biomarkers. SCAs1, 2, and 6 are autosomal dominant diseases, and MSA-C is a sporadic disease, each characterized by progressive gait ataxia, hand incoordination, dysarthria, and in many cases, a variable pattern of other progressive neurological deficits [3–8]. These symptoms are due to a loss of Purkinje cells within the cerebellum along with neuronal loss variably in other regions [9]. Recent developments have identified several genes and mutations specific for the different types of SCAs [3–5, 8]. SCAs are thus remarkably differentiated from other neurodegenerative diseases in that a specific genetic diagnosis can often be made, whereas in diseases such as AD, a definitive diagnosis cannot be made until autopsy. A specific genetic diagnosis ensures diagnostic and etiologic uniformity such that any changes in CSF protein composition can be more specifically attributed to a single disease cause unlike in PD or AD. In comparison to the SCAs, strict diagnostic inclusion criteria must be used for MSA-C [10, 11].

For this study, we selected several CSF biomarkers that have been well studied among neurodegenerative diseases. In patients with PD, *α*-synuclein is significantly decreased in the CSF and accumulates in the brain [12]. *α*-Synuclein, along with tau, is found in the glial cytoplasmic inclusions (GCIs) in the MSA-C brain, making it a prime biomarker candidate for this study [13, 14]. Tau protein promotes microtubule assembly and stability; its release is a marker of neuroaxonal damage in patients with neurodegenerative disease and is an established biomarker in Creutzfeldt Jakob disease and AD [2, 15]. Tau has also been shown to be predictive of the progression from mild cognitive impairment to AD, indicating the potential of biomarkers as a predictive tool [16–18]. DJ-1, also called PARK7, is a multifunctional protein involved in the regulation of oxidative stress, whose dysfunction can lead to cell death. In the early stages of PD, DJ-1 is elevated in the CSF [19]. Finally, glial fibrillary acidic protein (GFAP), a marker of glial cell activation, is involved in the communication between glial cells and Purkinje cells, the main cell affected in these disorders [20]. Elevated levels of GFAP have been shown in the CSF of AD and MS patients [2, 21]. A recent review of 60 papers on biomarker evidence in MSA was performed demonstrating generally decreased *α*-synuclein levels relative to controls, mixed results for tau with some evidence of increased levels while other showed no change, inconclusive results for DJ-1, and no change in GFAP levels relative to controls [14]. This demonstrates the continued need for reliable assessment of biomarkers and need for further studies which this pilot study aims to begin to address.

In another portion of this study, magnetic resonance spectroscopy (MRS) was performed to measure the level of metabolites in the brains of subjects with various SCAs and MSA-C relative to control subjects. This study showed alterations in neurochemical profiles of these neurodegenerative diseases and demonstrated the ability to use these biomarkers to differentiate between the ataxia subtypes [22]. Based on these studies, we tested the hypothesis that CSF neurochemical profiles of SCA1, SCA2, SCA6, and MSA-C will show distinctive qualitative and/or quantitative changes in CSF proteins that are involved in general neurodegeneration or in disease-specific processes.

## 2. Methods

### 2.1. Subjects

The sample cohort consisted of 5 SCA1, 6 SCA2, 5 SCA6, and 5 MSA-C patients, along with 5 controls (Table 1). Patients were recruited at the University of Minnesota Ataxia Clinic in 2005 and 2006, and control samples were collected from investigators and spouses of participating patients. Subjects had a confirmed genetic diagnosis of SCA1, SCA2, or SCA6, were diagnosed with MSA-C using established consensus criteria [10, 11], or were control subjects who had no neurological disorder. MSA-C patients were screened for all commercially available genetic forms of ataxia. Exclusion criteria included dementia or other neurological diseases.

### 2.2. CSF Collection and Storage

Sample collection was approved by the Institutional Review Board and was conducted in accordance with the Helsinki Declaration, and all CSF was collected on a research basis after obtaining informed consent. CSF was obtained by lumbar puncture (LP) under local anesthesia, with no side effects reported. CSF was centrifuged to remove cells, and supernatant was aliquoted and stored at −80°C.

### 2.3. CSF Protein Quantification

**Figure 1:**
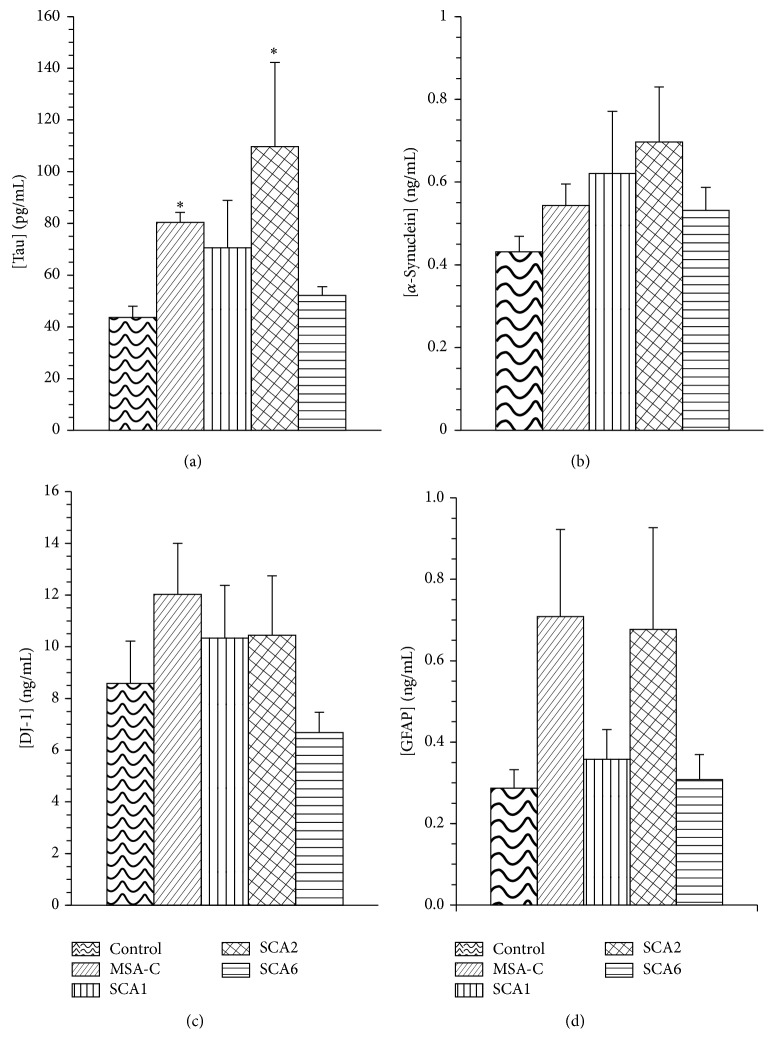
Biomarker concentrations by disease state. Mean calculated concentrations of (a) tau, (b) *α*-synuclein, (c) DJ-1, and (d) GFAP generated from the standard ELISA curve are shown with standard error bars. (*∗*) indicates *p* < 0.0125, significant after Bonferroni correction. Statistics calculated using Kruskal-Wallis ANOVA followed by post hoc Mann-Whitney *U* for disease state versus control.

Concentrations of *α*-synuclein, DJ-1/PARK7, tau, and GFAP were measured using enzyme-linked immunoabsorbent assays (Invitrogen KHB0061, MLB CY-9050, Invitrogen KHB0041, and BioVendor RD192072200R, resp.), according to the manufacturers' instructions. Average CSF concentration of each of the 4 biomarkers was calculated from the microplate reader generated standard ELISA curve and equation (Table 2, Figures 1(a)–1(d)) for each disease state. Within each ELISA quantification, samples were run in triplicate.

### 2.4. SARA Rating Scale

The standardized Scale for the Assessment and Rating of Ataxia (SARA) was used to evaluate the disease severity of the patients and to determine any correlation between biomarkers and variation in severity (Table 1). The scale ranges from no ataxia with a score of 0 to the most severe ataxia with a score of 40 [23, 24].

### 2.5. Expanded CAG Repeat Length

SCA1, SCA2, and SCA6 disease states were confirmed by genetic testing using a commercial assay of a panel of eight forms of SCA that demonstrated a CAG repeat expansion of one of the alleles for a given SCA type. Reported lengths are for the expanded allele alone (Table 1).

### 2.6. Statistical Analysis

Kruskal-Wallis nonparametric ANOVA was performed with follow-up Mann-Whitney *U* to determine differences in CSF levels of *α*-synuclein, DJ-1, tau, and GFAP in the 5 groups (control and 4 disease states) to account for small sample size and data of unknown distribution. Because of analysis of several different proteins, post hoc correction using Bonferroni method was employed, with a *p* value of 0.0125 determined to be the cutoff for statistical significance. SARA scores and CAG expansion length were correlated with the concentrations of biomarker levels using the Spearman correlation coefficient. Statistical calculations were performed using SPSSv17.

## 3. Results

### 3.1. Clinical Data

The mean ages of onset for the patient groups ranged from 33 years for SCA2 to 47 years for MSA-C, and the disease duration at time of study varied inversely from 8 years for MSA-C to 23.6 years for SCA2. The mean age at enrollment/CSF collection was similar between all patient groups and controls (Table 1).

SARA scores, a measurement of disease severity, ranged from 9.7 for SCA2 to 20.7 for MSA-C. Mean control SARA score was 0.375 (Table 1).

#### 3.1.1. CSF Levels

*Tau*. Average tau levels tended to be higher in all patients, ranging from 52.2 pg/mL for SCA6 to 109.7 pg/mL for SCA2 relative to control value of 43.7 pg/mL (Figure 1(a)). Kruskal-Wallis ANOVA indicated a significant effect of disease state on tau levels (*X* ^2^ = 15.9, *p* = 0.003). Post hoc Mann-Whitney *U* with Bonferroni correction showed that tau levels were significantly higher for SCA2 (109.7 ± 32.48 pg/mL, *p* = 0.009) and MSA-C (80.43 ± 3.84 pg/mL, *p* = 0.006) than for control (43.7 ± 4.34 pg/mL).

*α-Synuclein*. Although Kruskal-Wallis ANOVA indicated no significant effect of disease state on *α*-synuclein levels (*X* ^2^ = 6.3, *p* = 0.18), trends within the analysis were noted (Figure 1(b)). Average levels of CSF *α*-synuclein trended higher in all patients groups, particularly in SCA2 (0.70 ± 0.133 ng/mL), relative to control (0.43 ± 0.038 ng/mL), although the result did not reach statistical significance.

*DJ-1*. While the average CSF concentrations for DJ-1 (Figure 1(c)) did not vary significantly by disease state (*X* ^2^ = 6.7, *p* = 0.16), levels trended higher in SCA1 (10.3 ± 2.04 ng/mL), SCA2 (10.4 ± 2.30 ng/mL), and MSA-C (12.0 ± 1.97 ng/mL) versus control (8.6 ± 1.63 ng/mL). The mean CSF level of DJ-1 for SCA6 (6.69 ± 0.774 ng/mL) was actually lower than control, yet not significantly so.

*GFAP*. Mean CSF concentrations of GFAP (Figure 1(d)) tended to be higher for MSA-C (0.71 ± 0.214 ng/mL) and SCA2 (0.68 ± 0.250 ng/mL) than controls (0.29 ± 0.045 ng/mL) but the effect of disease state on GFAP level did not reach overall statistical significance (*X* ^2^ = 6.9, *p* = 0.143). No difference in GFAP levels was seen between the control population and either SCA1 patients (0.36 ± 0.073 ng/mL) or SCA6 patients (0.31 ± 0.061 ng/mL).

#### 3.1.2. Correlation with Disease Severity and CAG Repeat Expansion Size

We analyzed the effect of disease severity, as measured by SARA scores, on CSF biomarker concentration using a Spearman regression. Upon plotting individual biomarker levels as a function of SARA score, tau levels showed modest positive correlation, despite being only in MSA-C patients (*r* ^2^ = 0.617, *p* = 0.037). *α*-Synuclein levels correlated inversely with disease severity, despite being only in SCA1 patients (*r* ^2^ = 0.864, *p* = 0.01). Neither GFAP nor DJ-1 levels showed significant correlations with disease severity among any of the disease states (Table 2, Supplemental Tables 2–6 in Supplementary Material available online at http://dx.doi.org/10.1155/2015/413098).

There were no significant correlations between any of the biomarkers and the length of CAG repeat expansion as assessed by Spearman regression (Table 3).

## 4. Discussion

Biomarkers in neurodegenerative diseases have shown a growing potential for diagnosis and staging of diseases. In this preliminary study, we investigated whether there are differences in levels of certain proteins in CSF in clinically and genetically defined forms of spinocerebellar ataxia relative to healthy controls.

We found that tau levels were significantly elevated in both MSA-C and SCA2 patients relative to controls. The concentrations of *α*-synuclein, DJ-1, and GFAP in the CSF in SCA1, SCA2, SCA6, and MSA-C were not statistically different from controls, although consistent trends and patterns were present, indicating the potential for statistical significance in future studies with larger sample sizes and greater power.

Our findings concerning elevated tau in MSA-C patients are at odds with the decreased levels found in a previous work [25]. This discrepancy could be due to different stages of disease or differences in the specific subtype of MSA (MSA-C versus MSA-P). Glial cytoplasmic inclusions (GCIs) seen in MSA-C stain for tau, thus potentially indicating an overproduction of tau in these cells, which could be reflected in the CSF and explain our findings. Recent genetic studies have identified loss of function mutations in the gene CoQ2 in rare forms of MSA-C, indicating the existence of genetic subtypes of this disorder and the possible explanation for differing results [26]. The genetic data for these patients were collected well prior to this report. Increased CSF tau in SCA2 shows no known link to disease pathogenesis other than generalized neuroaxonal degeneration.

In our recent study of neurochemical changes in brain regions in SCA1, SCA2, SCA6, and MSA-C, we found MSA-C and SCA-2 had significant increases in markers of gliosis (myoinositol and glutamine), consistent with our finding of trends towards increased CSF GFAP in these diseases [22]. SCA2 was also the only autosomal dominant spinocerebellar ataxia investigated that showed tau elevation, consistent with the finding that SCA2 shows the greatest reduction in N-acetyl-aspartate (a neuronal marker) by MRS [22].

These findings may provide insights into the differential pathogeneses of these diseases. Additional studies may show that biomarkers can be used to improve understanding of disease pathogenesis and progression and potentially the identification of therapeutic targets. Finally, future studies could establish whether levels of one or more of these biomarkers, particularly if measurable in serum, might be monitored alongside the SARA score to predict progression and treatment efficacy.

While our current correlation statistics analyzing potential relationships between SARA score and CSF biomarker concentrations did not yield statistically significant findings, this is likely a result of decreased power secondary to small sample size and lack of broad range of disease severity. For example, SARA scores in the SCA6 group ranged from 14 to 18, covering a very limited range in the 0–40 scale. A larger cohort of patients representing a wider range of disease severity may provide a more complete cross-sectional study that would confirm the observed trends. Alternatively, serial analyses of these and other CSF proteins in individual patients may validate certain proteins as disease biomarkers.

## 5. Conclusion

In summary, there are quantitative changes in the CSF of ataxia patients not explained by variance in disease severity and duration. In both SCA2 and MSA-C, the level of tau is significantly higher than controls. Although no significant changes were seen in levels of *α*-synuclein, DJ-1, or GFAP, the levels of *α*-synuclein trended higher in SCA2. If replicated in a larger study, some of these or other CSF biomarkers might be used to identify disease pathogenesis and potential therapeutic targets and to monitor response to therapy.

## Supplementary Material

The supplemental tables provide additional analyses of the biomarker data. There is a breakdown of the average biomarker concentration of each of the four biomarkers in individual disease states, a tabular representation of Figure 1. Additional tables provide the data used to perform Spearman regression analysis. This analysis was performed to search for relationships between biomarker concentrations and SARA scores (a proxy for disease severity) in individual subjects.

## Figures and Tables

**Table 1:** Patient demographics.

Patient demographics					
	Control (*N* = 5)	MSA-C (*N* = 5)	SCA1 (*N* = 5)	SCA2 (*N* = 6)	SCA6 (*N* = 5)
Gender (F/M)	3/2	3/2	1/4	1/5	3/2
Age (mean ± SEM) [range]	50.8 ± 5.79 [34–70]	55.4 ± 1.50 [50–59]	52.0 ± 3.26 [43–61]	54.0 ± 1.85 [49–59]	53.4 ± 3.70 [45–66]
Age at onset (mean ± SEM) [range]		47.2 ± 3.89 [32–53]	41.2 ± 1.59 [38–47]	32.7 ± 3.70 [18–44]	37.6 ± 5.35 [25–55]
Symptom duration, years (mean ± SEM) [range]		8.2 ± 2.67 [3–18]	10.8 ± 2.22 [4–15]	23.6 ± 4.92 [11–40]	15.8 ± 2.58 [11–25]
SARA score (0–40) (mean ± SEM) [range]	0.375 ± 0.214 [0-1]	20.7 ± 5.12 [8–39]	11.1 ± 1.41 [6–14.5]	9.7 ± 1.23 [6–13]	15.9 ± 0.557 [14–17.5]
Expanded CAG repeat size (mean ± SEM) [range]			46 ± 1.58 [42–50]	37.5 ± 0.34 [36–38]	24 ± 1.29 [21–27]

Data are shown as mean (standard error). SARA score is a validated rating scale for the severity of ataxia ranging 0–40.

**Table 2:** SARA score, Spearman correlation coefficient, and *p* values.

Spearman correlation coefficient (*p* value):				
SARA score and biomarker concentration by disease state				
	Tau	*α*-Synuclein	DJ-1	GFAP
Control	0.105 (0.895)	0.105 (0.895)	0.105 (0.895)	−0.632 (0.368)
MSA-C	0.900 (0.037)^*∗*^	0.700 (0.188)	0.700 (0.188)	0.400 (0.505)
SCA1	−0.400 (0.505)	−1.00 (0.010)^*∗*^	−0.700 (0.188)	0.300 (0.624)
SCA2	0.058 (0.913)	−0.087 (0.870)	0.319 (0.538)	0.116 (0.827)
SCA6	−0.447 (0.450)	0.224 (0.718)	−0.671 (0.215)	−0.447 (0.450)

Spearman correlations were performed to elucidate any relationship between disease severity (as determined by an increased SARA score) and biomarker concentrations with each disease state. Data are expressed as correlation coefficient (*p* value). (*∗*) indicates *p* < 0.05.

**Table 3:** Expanded CAG repeat length, Spearman correlation coefficient, and *p* values.

Spearman correlation coefficient (*p* value):				
Expanded CAG repeat length and biomarker concentration by disease state				
	Tau	*α*-Synuclein	DJ-1	GFAP
SCA1	−0.700 (0.188)	0.100 (0.873)	−0.100 (0.873)	0.500 (0.391)
SCA2	0.304 (0.558)	0.439 (0.383)	0.304 (0.558)	−0.101 (0.848)
SCA6	−0.410 (0.493)	−0.718 (0.172)	−0.154 (0.805)	0.103 (0.870)

Spearman correlations were performed to elucidate any relationship between expanded CAG repeat length and biomarker concentrations within each disease state. Data are expressed as correlation coefficient (*p* value).

## References

[1] Dunckley T., Coon K. D., Stephan D. A. (2005). Discovery and development of biomarkers of neurological disease. *Drug Discovery Today*.

[2] Tumani H., Teunissen C., Süssmuth S., Otto M., Ludolph A. C., Brettschneider J. (2008). Cerebrospinal fluid biomarkers of neurodegeneration in chronic neurological diseases. *Expert Review of Molecular Diagnostics*.

[3] Banfi S., Zoghbi H. Y. (1994). Molecular genetics of hereditary ataxias. *Bailliere's Clinical Neurology*.

[4] Klockgether T., Dichgans J. (1997). The genetic basis of hereditary ataxia. *Progress in Brain Research*.

[5] Koeppen A. H. (2011). Friedreich's ataxia: pathology, pathogenesis, and molecular genetics. *Journal of the Neurological Sciences*.

[6] Manto M., Marmolino D. (2009). Cerebellar disorders-at the crossroad of molecular pathways and diagnosis. *Cerebellum*.

[7] Pandolfo M. (1999). Molecular pathogenesis of friedreich ataxia. *Archives of Neurology*.

[8] Schmitz-Hübsch T., Klockgether T. (2008). An update on inherited ataxias. *Current Neurology and Neuroscience Reports*.

[9] Konigsmark B. W., Weiner L. P. (1970). The olivopontocerebellar atrophies: a review. *Medicine*.

[10] Gilman S., Low P. A., Quinn N. (1999). Consensus statement on the diagnosis of multiple system atrophy. *Journal of the Neurological Sciences*.

[11] Gilman S., Wenning G. K., Low P. A. (2008). Second consensus statement on the diagnosis of multiple system atrophy. *Neurology*.

[12] Tokuda T., Salem S. A., Allsop D. (2006). Decreased alpha-synuclein in cerebrospinal fluid of aged individuals and subjects with Parkinson's disease. *Biochemical and Biophysical Research Communications*.

[13] Burn D. J., Jaros E. (2001). Multiple system atrophy: cellular and molecular pathology. *Journal of Clinical Pathology—Molecular Pathology*.

[14] Laurens B., Constantinescu R., Freeman R. (2015). Fluid biomarkers in multiple system atrophy: a review of the MSA Biomarker Initiative. *Neurobiology of Disease*.

[15] Blennow K., Hampel H. (2003). CSF markers for incipient Alzheimer's disease. *The Lancet Neurology*.

[16] Fagan A. M., Roe C. M., Xiong C., Mintun M. A., Morris J. C., Holtzman D. M. (2007). Cerebrospinal fluid tau/*β*-amyloid42 ratio as a prediction of cognitive decline in nondemented older adults. *Archives of Neurology*.

[17] Hansson O., Zetterberg H., Buchhave P., Londos E., Blennow K., Minthon L. (2006). Association between CSF biomarkers and incipient Alzheimer's disease in patients with mild cognitive impairment: a follow-up study. *The Lancet Neurology*.

[18] Roe C. M., Fagan A. M., Williams M. M. (2011). Improving CSF biomarker accuracy in predicting prevalent and incident Alzheimer disease. *Neurology*.

[19] Waragai M., Wei J., Fujita M. (2006). Increased level of DJ-1 in the cerebrospinal fluids of sporadic Parkinson's disease. *Biochemical and Biophysical Research Communications*.

[20] Shibuki K., Gomi H., Chen L. (1996). Deficient cerebellar long-term depression, impaired eyeblink conditioning, and normal motor coordination in GFAP mutant mice. *Neuron*.

[21] Malmeström C., Haghighi S., Rosengren L., Andersen O., Lycke J. (2003). Neurofilament light protein and glial fibrillary acidic protein as biological markers in MS. *Neurology*.

[22] Öz G., Iltis I., Hutter D., Thomas W., Bushara K. O., Gomez C. M. (2011). Distinct neurochemical profiles of spinocerebellar ataxias 1, 2, 6, and cerebellar multiple system atrophy. *Cerebellum*.

[23] Weyer A., Abele M., Schmitz-Hübsch T. (2007). Reliability and validity of the scale for the assessment and rating of ataxia: a study in 64 ataxia patients. *Movement Disorders*.

[24] Schmitz-Hübsch T., du Montcel S. T., Baliko L. (2006). Scale for the assessment and rating of ataxia: development of a new clinical scale. *Neurology*.

[25] Shi M., Bradner J., Hancock A. M. (2011). Cerebrospinal fluid biomarkers for Parkinson disease diagnosis and progression. *Annals of Neurology*.

[26] The Multiple-System Atrophy Research Collaboration (2013). Mutations in *COQ2* in familial and sporadic multiple-system atrophy. *The New England Journal of Medicine*.

