# Supplementary material for: Cerebrospinal Fluid Biomarkers in Spinocerebellar Ataxia: A Pilot Study
Source: Dis Markers. 2015 Jul 22;2015:413098. doi: 10.1155/2015/413098 (PMC4525756; doi:10.1155/2015/413098)
Supplement: Supplementary file 1 — The supplemental tables provide additional analyses of the biomarker data. There is a breakdown of the average biomarker concentration of each of the four biomarkers in individual disease states, a tabular representation of Figure 1. Additional tables provide the data used to perform Spearman regression analysis. This analysis was performed to search for relationships between biomarker concentrations and SARA scores (a proxy for disease severity) in individual subjects. [file 413098.f1.doc]

**Supplemental Tables**

Supplemental Table 1:

| **ELISA Biomarker Concentration By Disease State** | | | | | |
| --- | --- | --- | --- | --- | --- |
| **Disease State** | **n** | **α-synuclein Concentration (ng/mL)** | **Tau Concentration (pg/mL)** | **DJ-1 Concentration (ng/mL)** | **GFAP Concentration (ng/mL)** |
| **Control** | 5 | 0.4312 | 43.667 | 8.588 | 0.287 |
| **MSA-C** | 5 | 0.5431 | 80.429 | 12.024 | 0.709 |
| **SCA1** | 5 | 0.6209 | 70.635 | 10.332 | 0.386 |
| **SCA2** | 6 | 0.6965 | 109.705 | 10.442 | 0.677 |
| **SCA6** | 5 | 0.5315 | 52.171 | 6.688 | 0.308 |

**Supplemental Table 1. ELISA biomarker concentrations.** Patient samples were run in triplicate and the average fluorescence was used to calculate concentrations from the standard curve generated by the fluorescence microplate reader. SCA=spinocerebellar ataxia, MSA-C=multiple system atrophy of the cerebellar type.

Supplemental Table 2

| **Spearman Correlation Coefficient p-value:**  **Control SARA Scores and Biomarker Concentrations** | | | | | |
| --- | --- | --- | --- | --- | --- |
|  | **SARA score** | **average tau level** | **average a-synuclein level** | **average DJ-1 level** | **average GFAP level** |
| **control 1** |  | 45.48 | 0.433 | 7.35 | 0.373 |
| **control 2** | 0 | 53.97 | 0.556 | 14.6 | 0.417 |
| **control 3** | 0.5 | 38.34 | 0.397 | 7.55 | 0.242 |
| **control 4** | 1 | 50.68 | 0.446 | 8.64 | 0.192 |
| **control 5** | 0 | 29.87 | 0.323 | 4.8 | 0.21 |
| **Spearman correlation p-value** |  | **0.895** | **0.895** | **0.895** | **0.368** |

**Supplemental Table 2. SARA score and average biomarker concentration of controls.** Each control was evaluated using the SARA and results are shown, along with the average biomarker concentration for each individual. Spearman correlation was used to analyze any relationship between biomarker level and SARA scores in individual patients, and p-values are reported.

Supplemental Table 3

| **Spearman Correlation Coefficient p-value:**  **MSA-C SARA Scores and Biomarker Concentrations** | | | | | |
| --- | --- | --- | --- | --- | --- |
|  | **SARA score** | **average tau level** | **average a-synuclein level** | **average DJ-1 level** | **average GFAP level** |
| **MSA-C 1** | 8 | 71.53 | 0.423 | 8.26 | 0.253 |
| **MSA-C 2** | 16 | 71.9 | 0.494 | 9.07 | 0.586 |
| **MSA-C 3** | 19 | 80.92 | 0.736 | 19.36 | 0.761 |
| **MSA-C 4** | 39 | 88.59 | 0.532 | 12.38 | 0.448 |
| **MSA-C 5** | 21.5 | 89.2 | 0.53 | 11.05 | 1.497 |
| **Spearman correlation p-value** |  | **0.037*** | **0.188** | **0.188** | **0.505** |

**Supplemental Table 3. SARA score and average biomarker concentration of MSA-C patients.** Each MSA-C patient was evaluated using the SARA and results are shown, along with the average biomarker concentration. Spearman correlation was used to analyze any relationship between biomarker level and SARA scores in each individual patient and p-values are reported (* = p<0.05).

Supplemental Table 4

| **Spearman Correlation Coefficient p-value:**  **SCA1 SARA Scores and Biomarker Concentrations** | | | | | |
| --- | --- | --- | --- | --- | --- |
|  | **SARA score** | **average tau level** | **average a-synuclein level** | **average DJ-1 level** | **average GFAP level** |
| **SCA1-1** | 14.5 | 47.84 | 0.439 | 5.9 | 0.321 |
| **SCA1-2** | 11 | 44.87 | 0.494 | 9.75 | 0.578 |
| **SCA1-3** | 6 | 142.84 | 1.219 | 17.98 | 0.197 |
| **SCA1-4** | 12.5 | 57.94 | 0.468 | 9.79 | 0.47 |
| **SCA1-5** | 11.5 | 59.69 | 0.484 | 8.24 | 0.224 |
| **Spearman correlation p-value** |  | **0.505** | **0.010*** | **0.188** | **0.624** |

**Supplemental Table 4. SARA score and average biomarker concentration of SCA1 patients.** Each SCA-1 patient was evaluated using the SARA and results are shown, along with the average biomarker concentration. Spearman correlation was used to analyze any relationship between biomarker level and SARA scores in each individual patient and p-values are reported (* = p<0.05).

Supplemental Table 5

| **Spearman Correlation Coefficient p-value:**  **SCA2 SARA Scores and Biomarker Concentrations** | | | | | |
| --- | --- | --- | --- | --- | --- |
|  | **SARA score** | **average tau level** | **average a-synuclein level** | **average DJ-1 level** | **average GFAP level** |
| **SCA2-1** | 13 | 76.13 | 0.483 | 10.75 | 1.87 |
| **SCA2-2** | 6 | 80.43 | 0.518 | 10.07 | 0.758 |
| **SCA2-3** | 12 | 84.92 | 0.6 | 7.21 | 0.275 |
| **SCA2-4** | 7 | 79.74 | 0.676 | 7.49 | 0.33 |
| **SCA2-5** | 12 | 271.57 | 1.348 | 21.3 | 0.516 |
| **SCA2-6** | 8 | 65.43 | 0.555 | 5.83 | 0.313 |
| **Spearman correlation p-value** |  | **0.913** | **0.870** | **0.538** | **0.827** |

**Supplemental Table 5. SARA score and average biomarker concentration of SCA-2 patients.** Each SCA-2 patient was evaluated using the SARA and results are shown, along with the average biomarker concentration. Spearman correlation was used to analyze any relationship between biomarker level and SARA scores in each individual patient and p-values are reported.

Supplemental Table 6

| **Spearman Correlation Coefficient p-value:**  **SCA6 SARA Scores and Biomarker Concentrations** | | | | | |
| --- | --- | --- | --- | --- | --- |
|  | **SARA score** | **average tau level** | **average a-synuclein level** | **average DJ-1 level** | **average GFAP level** |
| **SCA6-1** | 16 | 58.93 | 0.617 | 7.25 | 0.216 |
| **SCA6-2** | 16 | 46.41 | 0.682 | 4.46 | 0.393 |
| **SCA6-3** | 17.5 | 48.96 | 0.51 | 5.96 | 0.121 |
| **SCA6-4** | 14 | 61.65 | 0.491 | 9.17 | 0.359 |
| **SCA6-5** | 16 | 44.89 | 0.357 | 6.6 | 0.453 |
| **Spearman correlation p-value** |  | **0.450** | **0.718** | **0.215** | **0.450** |

**Supplemental Table 6. SARA score and average biomarker concentration of SCA-6 patients.** Each SCA-6 patient was evaluated using the SARA and results are shown, along with the average biomarker concentration. Spearman correlation was used to analyze any relationship between biomarker level and SARA scores in each individual patient and p-values are reported.
